# Epileptic Patient Activity Recognition System Using Extreme Learning Machine Method

**DOI:** 10.3390/biomedicines11030816

**Published:** 2023-03-07

**Authors:** Ummara Ayman, Muhammad Sultan Zia, Ofonime Dominic Okon, Najam-ur Rehman, Talha Meraj, Adham E. Ragab, Hafiz Tayyab Rauf

**Affiliations:** 1 Department of Computer Science, The University of Lahore, Chenab Campus, Gujrat 50700, Pakistan; 2 Department of Computer Science, The University of Chenab, Gujrat 50700, Pakistan; 3 Department Of Electrical/Electronics & Computer Engineering, Faculty of Engineering, University of Uyo, Uyo 520103, Nigeria; 4 Department of Human Resource Section, Hafiz Hayat Campus, University of Gujrat, Gujrat 50700, Pakistan; 5 Department of Computer Science, COMSATS University Islamabad—Wah Campus, Wah Cantt 47040, Pakistan; 6 Industrial Engineering Department, College of Engineering, King Saud University, P.O. Box 800, Riyadh 11421, Saudi Arabia; 7 Centre for Smart Systems, AI and Cybersecurity, Staffordshire University, Stoke-on-Trent ST4 2DE, UK

**Keywords:** electroencephalography, deep learning, machine learning, epileptic seizure detection, human activity recognition, extreme learning machine

## Abstract

The Human Activity Recognition (HAR) system is the hottest research area in clinical research. The HAR plays a vital role in learning about a patient’s abnormal activities; based upon this information, the patient’s psychological state can be estimated. An epileptic seizure is a neurological disorder of the human brain and affects millions of people worldwide. If epilepsy is diagnosed correctly and in an early stage, then up to 70% of people can be seizure-free. There is a need for intelligent automatic HAR systems that help clinicians diagnose neurological disorders accurately. In this research, we proposed a Deep Learning (DL) model that enables the detection of epileptic seizures in an automated way, addressing a need in clinical research. To recognize epileptic seizures from brain activities, EEG is a raw but good source of information. In previous studies, many techniques used raw data from EEG to help recognize epileptic patient activities; however, the applied method of extracting features required much intensive expertise from clinical aspects such as radiology and clinical methods. The image data are also used to diagnose epileptic seizures, but applying Machine Learning (ML) methods could address the overfitting problem. In this research, we mainly focused on classifying epilepsy through physical epileptic activities instead of feature engineering and performed the detection of epileptic seizures in three steps. In the first step, we used the open-source numerical dataset of epilepsy of Bonn university from the UCI Machine Learning repository. In the second step, data were fed to the proposed ELM model for training in different training and testing ratios with a little bit of rescaling because the dataset was already pre-processed, normalized, and restructured. In the third step, epileptic and non-epileptic activity was recognized, and in this step, EEG signal feature extraction was automatically performed by a DL model named ELM; features were selected by a Feature Selection (FS) algorithm based on ELM and the final classification was performed using the ELM classifier. In our presented research, seven different ML algorithms were applied for the binary classification of epileptic activities, including K-Nearest Neighbor (KNN), Naïve Bayes (NB), Logistic Regression (LR), Stochastic Gradient Boosting Classifier (SGDC), Gradient Boosting Classifier (GB), Decision Trees (DT), and three deep learning models named Extreme Learning Machine (ELM), Long Short-Term Memory (LSTM), and Artificial Neural Network (ANN). After deep analysis, it is observed that the best results were obtained by our proposed DL model, Extreme Learning Machine (ELM), with an accuracy of 100% accuracy and a 0.99 AUC. Such high performance has not attained in previous research. The proposed model’s performance was checked with other models in terms of performance parameters, namely confusion matrix, accuracy, precision, recall, F1-score, specificity, sensitivity, and the ROC curve.

## 1. Introduction

Human Activity Recognition (HAR) is a widely studied and targeted research area focusing on human beings’ average daily activity recognition. Even though activity recognition has attracted many researchers and has been studied for a long time, researchers did not just stop at HAR; they further targeted the subtype of activity recognition which is aimed at the recognition of abnormal activity in patients. Furthermore, researchers are moving toward abnormal activity detection of patients or physically impaired people [1]. Abnormal activities are activities that are rarely performed and vary in their characteristics from other regular activities. These abnormal activities can be hand flapping, vomiting, fainting, headaches, epileptic activities, chest pain, abnormal heartbeat, falling backward, falling forward, etc. [2]. According to medical professionals, the best way to detect these activities is to look toward the emerging change in daily life activities before becoming more critical. In recent research, different methods have been used to detect abnormal activities, including wearable, sensor-based, and ambient device methods. The triggered alarm checks the status of activity detection. The detection accuracy of these activities depends upon analyzing and learning the patterns of exact features [3].

Although they solved this challenging problem by detecting abnormal activities of different diseases, they did not target the detection of epileptic disorders through epileptic activities. Many abnormal activities are mentioned above, but this study focuses on those observed during epileptic seizures. Very little work has been done on epileptic activity using a deep learning approach, and what has been published has not been in-depth. This study focuses on epilepsy detection through an epileptic activity using a deep learning approach. However, most of the work is conducted using medical imaging techniques that use images and not numerical data. Therefore, before digging deeper into previous detection methods, machine learning, or deep learning, we should have an idea about epilepsy.

Epilepsy is derived from the well-known Greek word “Epilepsia”. In ancient times when there was no well-known knowledge available about epilepsy, it was considered to be a curse from the gods. Furthermore, with time awareness about epilepsy grew and a method called Electroencephalography (EEG) was discovered. It is now understood that epilepsy is a strenuous, non-communicable, chronic, and uncontrolled activity in the brain of a patient and is associated with abrupt attacks that affect millions of people’s lives, be they adults, the elderly, or newborn babies. Epilepsy can occur at any age in both sexes, either male or female, but usually it is observed in people of a young or advanced age.

In the automated detection of epilepsy in previous research, the main focus is to differentiate between EEG and non-seizure EEG. Epileptic seizures are usually periods where the brain’s regular activity becomes abnormally increased and synchronized. Seizures are expeditious, and premature abnormalities in the brain due to anomalous electrical activity affect the person’s whole body. However, not all people who have seizures also have epilepsy because sometimes seizures can be caused by some psychological tissue stress in the brain; these are called non-epileptic seizures. As these non-epileptic seizures resemble actual seizures, this can make the diagnosis of the disease more complex [4].

Current epilepsy detection procedures assess the tissues of the brain that are generating epileptic seizure activity. There are many manual approaches as well as automated diagnostic methods that can be employed for detecting epilepsy. The most widely used traditional and manual method for epilepsy detection is through EEG. EEG represents a chaotic detection method because it produces blurred images as well as fails to represent the state and sites of the tissues of the brain [5]. The medical diagnosis of epilepsy is usually performed manually using EEG signals, which is very complex and requires highly skilled professional neurologists [6]. EEG signals are categorized into two types: (i) scalp EEG and (ii) iEEG. In scalp EEG, electrodes are placed on the scalp of the patient to capture seizure periods, but in the case of iEEG, the electrodes are directly placed on the surface of the brain to directly detect brain seizure activity from the cerebral part of the brain. As in the case of scalp EEG, it is very complex and time-consuming to capture brain activity from the scalp of a patient, Therefore, it is called a traditional or manual method of detecting epileptic seizure activity. Although EEG is a very common method, it still has some limitations in detecting epilepsy signals due to epilepsy’s chaotic behavior, and although highly skilled professional neurologists have extensive experience in visually detecting seizure activity from EEG data, such analysis still requires a lot of time.

EEG signals in nature are neither linear nor stationary; therefore, they cause difficulties in the manual monitoring and detection of normal and abnormal activities. Although it is not an efficient method [7], EEG is an effective tool for the evaluation as well as treatment of epileptic seizures. However, the drawback of EEG is that it requires highly skilled and professional neurologists to detect epilepsy correctly due to its complexity, and even for an experienced professional it is very time-consuming. Therefore, there is a need for a computer-based system, or Computer-Aided Diagnosis (CAD) tools, for the automatic detection and interpretation of epilepsy to overcome the drawbacks associated with the traditional EEG method [8].

Computer-Aided Diagnosis (CAD) tools are automated methods to detect epilepsy. These are a combination of image processing, artificial intelligence, and computer vision. These are the best systems and are designed to be cost-effective, fast, as well as effective at detecting abnormalities [9]. CAD tools are proposed to be the best way to detect epileptic seizure activity and to perform feature extraction and classification from images to monitor the abnormal brain activity in a short period. In the field of medical imaging, CADe and CADx are helpful for medical professionals to make quick decisions. CAD tools use Magnetic Source Imaging (MSI), Magnetic Resonance Imaging (MRI), Magnetoencephalography (MEG), CT scans, etc. For the evaluation of epilepsy, the required data for these methods are scanned images. These are neuroimaging techniques for the evaluation of epilepsy that map brain electrical activities with detailed images produced using magnetic fields, radio waves, and electrical currents by interacting and recording seizures that are naturally present in the brain. These combined graphical, electrical, and structural data play an important role in the final selection of tools chosen for the evaluation of epilepsy, which further influences the final decision on treatment [10].

Furthermore, there are many signal- and image-processing-based procedures used for the evaluation of epilepsy with some spatial as well as temporal features. These tools can be used if there is a need for high-quality images for the evaluation of epileptic seizure activity, but these imaging techniques are very high energy and can thus harm the human body. Hence, these images are taken with less energy, due to which they are of bad quality, and later, the image quality is cost-effectively improved through CAD tools. This process helps to efficiently interpret the image with better accuracy and highlight conspicuous parts of the brain to detect epileptic seizure activity [11]. EEG is a necessary step for detecting epileptic seizures and it assists medical professionals in speeding up the detection process. For this purpose, along with CAD tools, many signal processing and classification techniques have been used in manual procedures but they still have some complexity, performance, classification, and speed issues [12]. Therefore, to overcome these problems, there is a need to use efficient machine learning algorithms for the proper classification of multi-class seizure activities. ML algorithms help to pre-process the images as well as to extract features. ML algorithms help to account for the state of the brain as well as predict epileptic seizures [7].

Recent research has shown that many ML algorithms assist neurologists in detecting epileptic seizures and classifying epileptic seizure activities. These include Support Vector Machine (SVM), K-Nearest Neighbors (KNN), Random Forest (RF), Neural Networks (NN), Naïve Bayes (NB), Logistic Regression (LR), Decision Trees (DT), Probabilistic Neural Networks (PNN), and Multilayer Perceptron (MLP) [13,14]. Although many of these machine learning algorithms are very successful in detecting epileptic seizure activities, they are still not as successful as Deep Learning algorithms. EEG signal analysis is very complex and requires a lot of human skill and expertise. Human work can be prone to errors in epilepsy detection. To overcome this issue, ML algorithms are used.

Although ML algorithms are helpful in overcoming human error, this problem is more tricky and complex than it looks; hence, somewhat more robust algorithms such as Deep Learning methods are required for epileptic seizure activity detection [15]. To automatically detect epilepsy, Deep Neural Networks are used that are based on the processing techniques of signals (EEG) as well as pattern recognition. They use the data of EEG for the detection of locations as well as durations of spikes and seizures. Recent advances in DL algorithms have paved the way for epileptic seizure detection. These include Artificial Neural Networks (ANNs), Convolutional Neural Networks (CNN), Long Short-Term Memory (LSTM), Hopified Networks (HN), Restricted Boltzmann Machine (RBN), and the Wavelet-Based approach. In our presented research, we applied an Extreme Learning Machine (ELM) for this purpose and also applied some ML techniques including KNN, NB, LR, DT, RF, GB, and SGDC, as well as the DL LSTM technique. A further review of recent studies is continued below.

There have been many types of research carried out in the field of HAR that combine different techniques which could be used for recognizing human activities. Recent research has shown that the recognition of activities is usually addressed by machine learning techniques including decision trees, Support Vector Machines, Bayesian Methods, Neural networks, fuzzy logic, Markov Models, Hidden Markov Models, and Regression models. Different authors have proposed different machine learning techniques for the recognition of normal and abnormal human activities. Normal activities include walking, standing up, sitting down, jumping, and eating. One author applied state-of-the-art techniques by using 2D AbHAR and 3D AbHAR datasets along with Hidden Markov Models (HMM) and KNN to recognize abnormal human activities such as chest pain, headaches, and fainting [1]. This author also applied Decision Trees, Support Vector Machine, and Hidden Markov Models to recognize abnormal activities such as walking to a chair, crouching, falling to the right or left, and falling forward and backward. This process was completed in two stages, namely data input through sensors and data processing, and recognized activities with 98% accuracy [16].

Although there are many kinds of research on normal human activity recognition, in recent years, there a lot of research is also being carried out on the recognition of activities of people who are physically impaired or injured ambulatory patients at home or who have psychological or neurological disorders. For security reasons, the recognition of abnormal activities has become an important and challenging task. The activities of such patients deviate from normal behavior and are usually referred to as abnormalities. Abnormal patient activities are those that require medical help immediately. The current research aims to effectively create new techniques as well as solutions for further research. To recognize abnormal patient activities and to monitor patient care either in the hospital or at home, different authors have proposed different machine learning techniques.

### 1.1. Machine Learning Techniques

One author used different machine learning techniques, namely K-Nearest Neighbors (KNN), Support Vector Machine (SVM), Decision Tree (DT), Artificial Neural Network (ANN), and Principal Component Analysis (PCA), to perform classification on an epileptic seizure dataset using 9200 normal activities named Partial epilepsy without seizures and 2300 abnormal epileptic activities named General epilepsy with seizures. The accuracy of the classifiers in the prediction of epilepsy using PCA and without using PCA was observed. Findings showed that RF without using PCA with low computational time and with 97% accuracy produced the best result. Without using PCA, KNN and RF achieved 99% accuracy as compared to other ML classifiers [17].

Another author used different ML algorithms, namely KNN, SVM, PCA, and ANN, for the recognition of the epileptic seizure dataset. This research had two principal methods. In the first method, features were extracted and then classified into binary classes labeled as Epileptic and Non-Epileptic seizures. In the second method, the performance of the feature extraction method was improved by using the Principal Component Analysis (PCA) method, and a 96% accuracy was achieved [18].

Another author used the SVM and its twin variant and then embedded these with univeram data to classify the EEG Signals. The author used Principal Component Analysis (PCA), Independent Component Analysis (ICA), and wavelet analysis. The author used 30 EEG datasets, one of which was the Bonn university EEG dataset which showed that accuracy was 100% [19].

Another author used different classifiers of machine learning to perform the classification of normal activities, named Tumor, Hstumor, Eyec, Eyeo, and abnormal activities, named Es, using the epileptic seizure dataset. The ML classifiers used for the classification were KNN, Logistic Regression, Naïve Bayes, Decision Tree, Stochastic Gradient Descent, J48, Random Tree, and Random Forest. Compared to the other ML classifiers, Random Forest greatly outperformed all of these classifiers and achieved a 97.08% maximum accuracy with an ROC = 0.996. In this research, to analyze the performance of different classifiers, a sensitivity analysis was performed by changing different parameters and using different attributes chosen as feature selection in prediction [20].

Another author proposed a model which used wavelet transform as well as common spatial pattern filtering for the pre-processing. For the purpose of feature extraction, the technique Principal Component Analysis (PCA) was used and for the Pre_ictal stage classifier, Support Vector Machine (SVM) was used, achieving an average sensitivity of 93.1% observed across 84 seizures in 23 subjects. The normal activities used in this paper were Pre_ictal, Inter_ictal, Eyes open, and Eyes Close, and the abnormal activity was Ictal [21].

Another author performed classification on the Epileptic Seizure dataset. The classifier which was used for classification was SVM, and the Bayesian optimization algorithm was used by the SVM classifier for the optimization of the hyperparameter. In this research paper, to perform the comparison, Linear Discriminant Analysis (LDA) and Quadratic Linear Discriminant Analysis (QLDA) were used, and the accuracy achieved by the SVM classifier was 97.05% [22].

Another author applied the Discrete Wavelet Transform (DWT) technique for feature extraction and then used the Radiant Basis Kernal function (RBF) to train the SVM classifier. For better EEG classification, an optimizer named the Grey Wolf Optimizer (GWO) was used for the selection of the important subset of features and parameters of the SVM with 99% accuracy [23].

Another author used the DWT technique to retrieve statistical features from the decomposition of EEG data into bands and the classifier was trained to learn these statistical features. To predict whether signals were epileptic or not, two classifiers named Naïve Bayes and KNN were used for the classification of the epileptic seizure dataset with normal epileptic activities named Eyes open, Eyes close, Inter_ictal, and Pre_ictal and abnormal activity named Epileptic seizure. In this research paper, 14 successful results of a different combination of two classes of epilepsy detection were obtained. A Naive Bayes classifier with less computing time and 100% accuracy was used for the classification of an epileptic dataset [24].

Another author used a classifier named dual-tree wavelet complex transform (DTCWT) for signal decomposition as well as for the calculation of statistical measurement. Then, for the training of the statistical environment, a classifier named General Regression Neural Network was used and achieved 95.24% maximum accuracy in less than 0.028 s [25].

Another author in his paper proposed a new method for epileptic and non-epileptic seizure detection. The transformation of the multiresolution decomposition was performed on the base of the wavelet and was then used in combination with an Artificial Neural Network (ANN) for the classification of the EEG signal regarding whether a seizure is present or not. The achieved accuracy was 97.77% using normal epileptic activities named Normal, Non-seizure, Non-seizure with eyes closed, and Non-seizure with eyes open and the abnormal activity named Seizure [26].

Another author used the technique of permutation entropy to extract the features from the signals of the EEG. For the classification purpose, the Support Vector Machine (SVM) classifier achieved the maximum accuracy of 93.55% for the selected case of A-E and the accuracy achieved for other datasets was 86.1% using normal activity named Tumor, Non-epileptic, Eyes close, Eyes Open, and abnormal activity named Seizure Activity [27].

Another author used DWT for the extraction of energy features, standard deviation, and entropy from the signals of the EEG. The maximum accuracy, i.e., 95.44%, was achieved by using the classifier Probabilistic Neural Network (PNN) and Support Vector Machine (SVM) and using normal activities named Tumor, Non-epileptic, Eyes close, and Eyes Open and abnormal activity named Seizure Activity [28].

Another author applied a patient-specific methodology and for classification applied an SVM classifier to distinguish between epileptic or non-epileptic activities of a patient. A maximum accuracy of 96% was achieved using normal epileptic activities named Eyes open, Eyes close, Inter_ictal, and Pre_ictal and abnormal activity named ictal [29].

Another author used Linear Discriminate Analysis (LDA) to classifying seizures using the data of 65 seizures across five patients and achieved 91.8% accuracy by using binary case classes in which the normal activity class was named Non-Epileptic Seizure and the abnormal activity class was named Epileptic-seizure [30].

Another author performed the detection and analysis of EEG data using entropies as well as seven different classifiers. The classifier which performed the best was the Fuzzy Sugeno classifier with an overall accuracy of 98.1%, a 100% specificity, and a 99.4% sensitivity. The classifier which performed the worst was Naïve Bayes, which achieved an accuracy of 88.1% using the normal activity classes named Normal and Pre-ictal and the abnormal activity class named Epileptic [31].

### 1.2. Deep Learning Techniques

Machine learning is very successful in the recognition of abnormal activities of patients. Therefore, there is a need for the automatic recognition of abnormal activities. For this purpose, different authors have proposed different deep learning algorithms that greatly help with automated detection. All the above-mentioned machine learning techniques did not use the automated method to extract features; instead, they used hand-crafted methods. Therefore, to avoid the extra effort of extracting features through the handcrafted method, deep learning techniques need to be used and DL models need a lot of data to train the DL models. In this section, we list a few researchers who used deep learning techniques to classify whether the activity is epileptic or non-epileptic. For example, one author proposed a deep learning model named the pyramidal One-Dimensional Convolutional Neural Network. This CNN model uses refined and less trainable parameters as compared to the traditional convolutional neural network. Compared to the state-of-the-art techniques, this model achieved an accuracy of 99.1% with normal activities named Eyes Close, Eyes Open, and Pre-ictal and abnormal activities named Inter-ictal and ictal [32].

In this research, the author performed epileptic seizure recognition through binary classification using classes labeled as Epileptic Seizure and Non-Epileptic Seizure. For the classification of monitored data, a two-layer Recurrent Neural Network (RNN) was used, and for the first layer and the dropout layer, Long Short-Term Memory (LSTM) and the Horse Optimization Algorithm (HOA) were used, respectively [33].

Another author used a one-dimensional, sequential Convolutional Neural Network (CNN). The architecture used for the classification of time series data of EEG comprised a one-dimensional module and a one-dimensional ResNet module to check whether there was epilepsy or not, achieving an AUC of 0.98 [34].

Another author proposed a deep learning model named Long Short-Term Memory (LSTM) for the training, detection, as well as prediction of epileptic seizures with state change as well as EEG seizures of a chaotic nature. The purpose of this research was to launch a small and low-cost gadget that can be wearable. Normal activities named Tumor, Hstumor, Eyec, and Eyeo and abnormal activities named Es on the Epileptic seizure dataset were used in this paper, achieving an accuracy of 99% [35].

Similarly, another author proposed a novel deep-learning-based model named Deep Canonical Sparse Autoencoder Epileptic Seizure Detection for the classification of EEG signals, which involved two steps, namely, feature selection and classification. This research involved a coyote algorithm for the selection of features and a novel derived classifier named DCSAE, based on an epileptic seizure detection model, for the classification of epileptic and non-epileptic seizures. The DSCAE was tuned by an algorithm named the krill herd algorithm (KHA). The maximum accuracy achieved in this research for binary classification was 98.67% and was 98.73% for multi-classification. Binary classification involved labelling abnormal activity (that is, EEG signals showing seizure activity) as 0 based on 2300 instances to train the model and labelling normal activity (that is, EEG signals having no seizure activity as 1 based on 9200 instances to train the model. Multi-classification involved labelling normal classes from EEG signals showing seizure activity, tumor regions, a healthy brain, eyes closed, and eyes open as 0, 1, 2, 3, and 4, respectively, with each class having 200 instances to train the model [36].

Another author proposed a DL-based model named EESC, abbreviated for Epileptic EEG signal classification. For the extraction of features, a DCNN, abbreviation for Deep Convolutional Neural Network, and the Transfer Learning method were used. In the end, four classes of normal activities were categorized as seizure and classes of abnormal activities were categorized as Inter-ictal and pre-ictal duration. An accuracy of 90% [37].

Many diseases such as Parkinson’s, Alzheimer’s, and epilepsy are the causes of stress or mental disorders. Hence, to monitor the activity of such types of diseases, different authors have proposed different deep learning techniques. For example, for monitoring patient activities, on author proposed a DL model for the detection of epileptic seizures on the basis of inter-ictal recordings, and on that data, filtration as well as segmentation was performed. In this research, Long Short-Term Memory (LSTM) as well as a Convolutional Neural Network (CNN) were used for the classification of the epileptic and non-epileptic data, and an accuracy of 94.74% was achieved [38].

Another author proposed an Artificial Neural Network (ANN) based on a deep learning model to solve the complex problem of detecting epileptic seizures. The deep learning CNN provided excellent accuracy on the EEG dataset. While many other ML techniques have been applied in combination to reduce and pre-process the data, CNN does not require pre-processing and achieved an accuracy of 95.24% using the normal activities named Pre_ictal, Inter_ictal, Eyes open, and Eyes Close and Ictal used as the abnormal activity [39].

Although this literature review has briefly described many types of research along with their benefits, there is still some room to improve the efficiency of classifying epileptic activity. Due to the incessant deepening of DL models, there has been a rapid increase in the number of parameters, which may in consequence lead to the problems of overfitting and generalization; therefore, there is a need to tune these parameters. Hence, to tune the parameters, there are also some hyperparameters available that affect the operation and efficiency of the Convolutional Neural Network (CNN). Usually, the hyperparameters that are effective and beneficial are batch size, learning rate, and the number of epochs. The trial-and-error method is the state-of-the-art method for checking every model’s performance, but it is an erroneous as well as very tedious process. Hence, to avoid this hectic and time-consuming process, metaheuristic algorithms can be used. Therefore, in our research, we used the DL technique named Extreme Learning Machine (ELM). Previous authors compared the results with the traditional method of EEG. However, in this paper, we detect epileptic seizures through patient activities observed during epileptic seizures.

### 1.3. Contribution

The main contribution of this research is that it does not use imaging data to train the classifier, and this is help to use less memory because of using numerical datasets and proposed model does not require backpropagation and calls for less user intervention.

We also tried to overcome the pressing problems of generalization and overfitting previous researchers faced by using the ELM classifier.

An efficient system with less training and testing time as compared to previous models is developed. It show improvements in terms of faster convergence and higher accuracy.

## 2. Proposed Methodology

**Figure 1:**
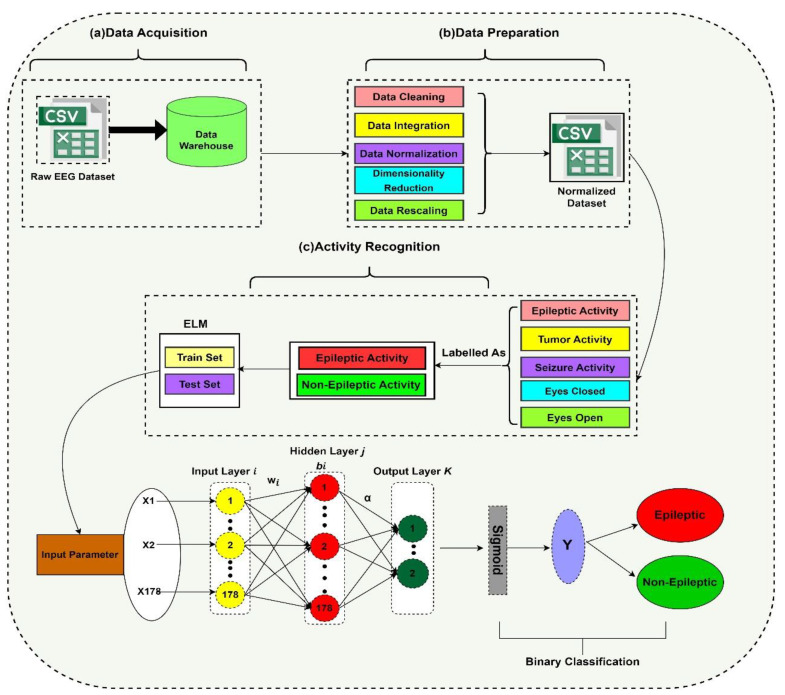
Proposed Methodology.

The proposed methodology consists of three steps: (i) Data Acquisition (ii) Data Preparation, and (iii) Activity Recognition. These three steps are described below, and the methodology is shown in Figure 1.

### 2.1. Data Acquisition

Data acquisition is a process to gather and store digital or numerical data to input into our model either in raw or pre-processed form, depending on the availability of data. Therefore, before data acquisition, it is very necessary to have an understanding of the dataset used.

- Dataset Understanding

**Figure 2:**
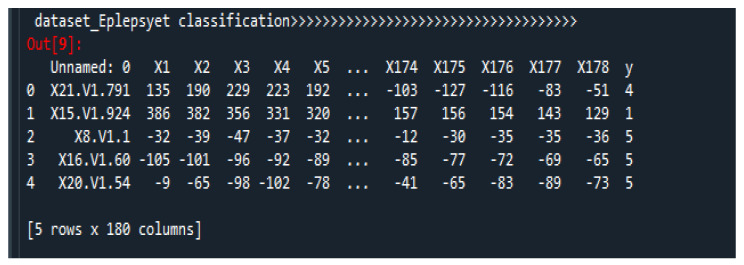
Randomly Sample View of Epileptic Recognition Dataset.

We used a numerical database named Epileptic Seizures Recognition Dataset, which is a pre-processed and well-organized dataset commonly used for the recognition of epileptic seizures provided by the UCI Machine Learning Repository. For epilepsy analysis and diagnosis, there are a lot of EEG datasets available on the Internet; some of these datasets are private due to a lack of ethical clearance, but some of these are made openly available by medical analysts for research purposes. The concerned dataset is openly and freely available from the UCI Machine Learning Repository provided by Bonn University [40]. As the current authors did not perform experiments on humans, animals, or plants to collect the data and instead made use of a freely and openly available dataset, this research does not require any ethical clearance. Based on this dataset’s description, this is the original dataset and it consists of 5 different folders. Each folder consists of 100 files, and each file represents an individual person’s information. In each of these files, brain activity was recorded for 23.6 s. The recorded brain activity is further sampled into 4097 different data points. Each recorded EEG value represents a data point at different points in time. Therefore, we have data from 500 individuals in total and each individual has 4097 data points over 23.5 s. All 4097 data points are further subdivided and shuffled into 23 different chunks. Each chunk further consists of 178 data points lasting 1 s. Each of these data points is the recorded value of the EEG at different points in time. Therefore, in total, we have 500 × 23 = 11,500 rows (instance) as a piece of recorded information. The random sample view of the Epileptic Seizure Recognition Dataset is shown in Figure 2.

In this dataset, 178 columns are used as a numerical input parameter and the last one, column 179, is used to label the data as output 1, 2, 3, 4, or 5. These five classes in the epileptic dataset are the five mentioned health conditions of epileptic patients in which one is the epileptic patient condition and the other four mentioned are normal conditions in which the concerned data point does not show epileptic activity. The details of all five classes and their samples are listed in the following Table 1.

**Figure 3:**
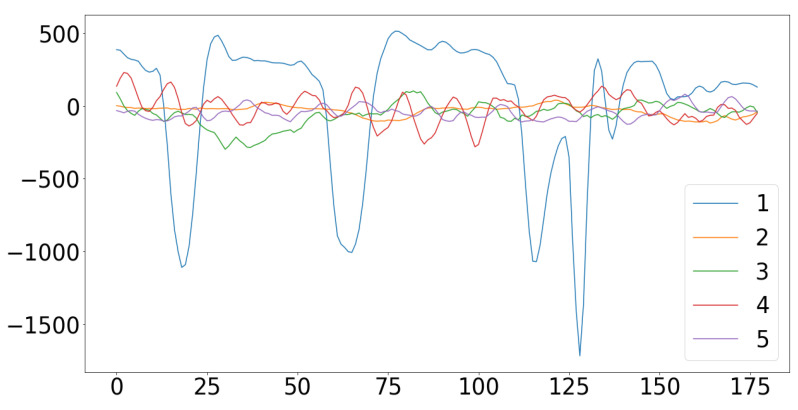
EEG Waveform of four normal conditions vs. one epileptic condition.

The raw EEG signal data of all five of the above-mentioned health conditions are shown as waveforms in Figure 3. This is the initial raw form of the data, but these data are not directly fed into the model. Although this dataset is a normalized, structured, and reshaped version of the data, the dataset needed to be rescaled into unit variance and the five classes needed to transform into binary form. Hence, data in their initial forms will not show the same result, which is not as promising as it requires pre-processing. These results will definitely change once the initial signal samples undergo pre-processing and preliminary transformation or if signal samples other than initial data are used. Hence, in this first stage of data acquisition, numerical recorded data are stored in an Excel spreadsheet used as a database from where data are read from columns X1 to X178 as input parameters to train the model, and the last column, named as Y, is used as an output column of labeled data. After reading the data from the database, the data are passed on to the next phase of data preparation according to the proposed model.

### 2.2. Data Preparation

Data preparation is a very complex phase of data mining in a project. The performance and results of the selected algorithm and model are highly impacted by the dirty and noisy dataset selected. A noisy dataset consists of data in which some significant values are missing as well as some outliers are present which may adversely result in low-quality and inconsistent data. Inconsistent data lead to unimpressive and invaluable results in the results evaluation phase. Therefore, data preparation plays an important role in the case of creating quality data; it is the phase in which data are manipulated and cleaned for the rest of the project phases. The principal tasks of data cleaning include data cleaning, data integration, data normalization, dimensionality reduction, and data splitting. However, since the UCI epileptic dataset is a normalized, pre-processed, structured, and reshaped version of the data. This dataset does not have any missing values or repetitive values.

### 2.3. Activity Recognition

**Figure 4:**
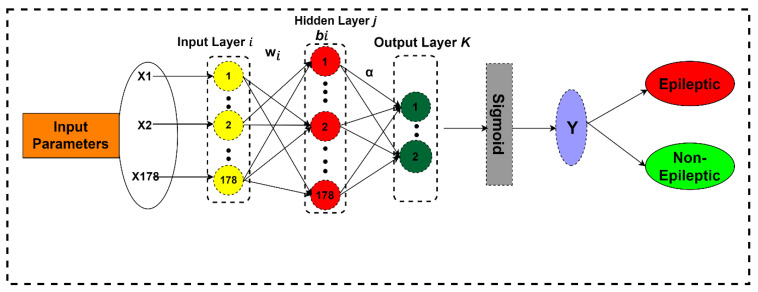
Extreme Learning Machine.

Activity recognition is a process of recognizing the performed activities read from the database after data preparation. These activities are represented in the form of different classes. There are 5 classes, and each class represents different activities: Seizure activity, Tumor activity, EEG activity, Eyes closed, and Eyes open. Although there are a lot of techniques of deep learning that can be applied to this complex recognition task, here we will use the Extreme Learning Machine (ELM) to train our model on these 5 epileptic activities by using the ratio of 80% training data and 20% testing data since ELM has a simple and straight-forward architecture and does not need any gradient-based propagation to work. The architecture of ELM is displayed in Figure 4.

Here, the ELM classifier is presented. ELM is a feedforward neural network and has one input layer i, a single hidden layer j, bias b, input weights w, and a single output layer k which is used at the end for the classification and activity recognition process.

The classifier used in this step is ELM and before digging deeper into the detail of the activity recognition process we will discuss the working of the ELM algorithm described below:

#### 2.3.1. Extreme Learning Machine (ELM):

According to the architecture of ELM shown in Figure 4, ELM is a feedforward neural network with a single hidden layer that selects the inputs and weights randomly and analytically determines the output weights of single hidden-layer feedforward networks (SLFNs) [41,42]. The most important key principle of ELM is that one can randomly choose or fix the hidden parameters of the node. After randomly selecting the hidden node parameters, the weights of outputs can be determined analytically by using hidden layer matrices for output by using generalized inverse operation when SLFN becomes the linear system [43]. In the hidden observation layer, the activation function Q is a sigmoid function and the *N* is the number of nodes of the data set; the extreme learning model can be expressed as:

(1) $$f(x)=\sum_{i=1}^{N}\alpha_{i}Q(w_{i},\;b_{i},\;x_{i})=\alpha \;\cdot \;h(x),\;$$

In the above equation, α*_i_* is the weight of the output neuron of the *i*th hidden layer node, whereas w*_i_* is the input weight of the input neurons of the *i*th hidden layer node; *b_i_* is the offset, usually called the bias, of the *i*th hidden layer node; and h(*x*) = [Q(w_1_, *b*_1_, *x*_1_),… Q (w_N_, *b*_N_, *x*_N_)] represents the hidden layer output matrix. Before training the algorithm, w*_i_* and *b_i_* are selected randomly and remain frozen during all training procedures. By solving the least-square solutions of the following linear equation, the output of weight α*_i_* can be obtained:

(2) $$min\sum_{i=1}^{N}\left|\left|\alpha_{i}\cdot h\left(x_{i}\right)-y_{i}\right|\right|$$

The least-square solution of the above equation is:

α = *H*^+^
*Y*, (3)

In the above equation, *H*^+^ is called the Moore–Penrose generalized inverse of the hidden layer output matrix *H*.

After analyzing the workings of the ELM, we will recognize the epileptic and non-epileptic activities through ELM. The principal steps to perform signal classification for the activity recognition process are discussed in the following sections.

#### 2.3.2. Feature Extraction

Before performing deep classification, first we need to extract the features to train the model. Keenly observing the previous research before the rise of DL shows us that manual feature extraction was performed by the conventional ML algorithms, and this use of ML algorithm was the reason the performance of the model’s ability was limited using handcrafted features. After the advent of DL, the process of feature extraction performed by the model became automated. In our proposed model, we just list all the features from X1 to X178 as the input feature data and Y as the target label data and then ELM will automatically reveal the correlations between the samples of successive data and will extract high-level representations of non-epileptic and epileptic signal features, resulting in a feature matrix. The learning of the extracted features by the model is performed through training by fitting the model using processed, extracted training input data at a ratio of 80% training data to 20% testing data.

#### 2.3.3. Feature Selection

The extracted features are then selected by the Feature Selection (FS) algorithm based on ELM. The working of the FS algorithm is described below:

- ELM-based Feature Selection Algorithm:

The neuron of the input layer’s contribution to the neuron of the output layer is reflected to some extent by the magnitude of the input weight. After the training of the ELM model is completed, the input weight’s and output weight’s information reflect the input feature’s importance. The ELM-based selection algorithm’s steps are described below:

Step No 1: In this step, we will calculate the significant coefficient correlation:

(4) $${c_{i}}_{j}=\sum_{i=1}^{q}\alpha_{jk}\;(1-e^{-w_{ij}})/1+e^{-w_{ij}})$$

Step No 2: In this step, we will calculate the index correlation:

(5) $${c_{i}}_{j}=|(1-e^{-c_{ij}})/1+e^{-c_{ij}})|\;$$

where $w_{ij}$ is the input weight used to connect the ith input layer nodes to jth hidden layer nodes and $\alpha_{jk}$ is the output weight used to connect the jth hidden layer node to the kth output layer node.

Step No 3: In this step, we will calculate the coefficient of absolute influence:

(6) $${S_{i}}_{j}=c_{ij}/\sum_{i=1}^{m}C_{ij}$$

Step No 4: In this step, we will calculate the feature weight:

(7) $${W_{i}}_{j}=\sum_{i=1}^{m}C_{ij}/m$$

By using this algorithm, the features selected are the features that have high weights; features which have low weights are not important and should be removed.

#### 2.3.4. Classification

Here, the classification of activities is based on the ELM classifier and works as shown in Figure 4. The model parameters’ input size is 178 neurons, and the hidden layer size of the neuron depends upon the input layer and is selected randomly. Therefore, firstly, the bias and weight matrix for the input layer of the model are created randomly. In the next step, the output matrix for the hidden layer is calculated by multiplying the training data (X) with the transposed weight matrix from the input layer. Here, the activation function used is Sigmoid because it yields better nonlinear transformations and feature mapping. In addition, it is easy to implement and its outcome has the best accuracy compared to other activation functions. In the next step, the Moore–Penrose pseudo-inverse is calculated. After that output weight matrix is calculated. After that, the result matrix is calculated, which is the epileptic activity as an output. We will use a trial-and-error strategy here to identify the best tuning parameters and optimize the model. We perform the classification and recognition of activities of epileptic disorders. By using binary classification, testing data will be further used for evaluating whether the 5 activities that are further binary labeled are accurately recognized or not. Here, prediction is performed by Predict(X), which is further used to give the target output (Y) when an unlabeled observation (X) is given as an input to the model.

## 3. Results and Discussion

### 3.1. Running Environment

The pipeline of our proposed methodology model is carried out with advanced and modern computer software tools and libraries. All working experiments are conducted on a system with an Intel Corei7 with 8 GB Ram and the Windows 10 operating system installed on it. The platform tool used for the experimentation and analysis of the results is Anaconda with python language version 3.8.6 installed. The Interactive Development Environment (IDE) used by Anaconda for the whole process of execution is Spyder.

### 3.2. Dataset Specification

The proposed model is applied to the UCI machine learning dataset consisting of five classes, which are later labeled as y = {0, 1}. 

0—Not Recorded Seizure;

1—Recorded Seizure.

The specifications for this dataset is briefly described in Table 2. 

The epileptic seizure dataset is divided into the following training and testing ratios to train the model for the best accuracy:

80% training, 20% testing;

70% training, 30% testing;

60% training, 40% testing.

### 3.3. Parameter Tuning

Parameter tuning is the procedure in which different parameters are set to tune the model to increase its performance. There are different parameters shown in Table 3, and their values increase the ELM model’s performance. The results of the model vary as the values of these parameters change.

To adequately evaluate the performance of the proposed model, the proposed deep learning model was trained using the three different dataset ratios and its performance measured in terms of F1-score, accuracy, specificity, sensitivity, precision, the area under the curve, and the ROC with some sufficient feature details. The experiments and the results of our proposed model are discussed in the remainder of this section.

### 3.4. Experiment 1

The Extreme Learning Machine (ELM) model was trained the best on 80% of data chosen randomly and the remaining 20% of data set aside for later use, i.e., for the evaluation of model accuracy, called testing, and for validation. The training accuracy of our model for this dataset ratio is 99.9% and the accuracy of testing is 100%, which is higher than the other dataset division ratios. Details of the ELM experiment are shown in Table 4.

**Figure 5:**
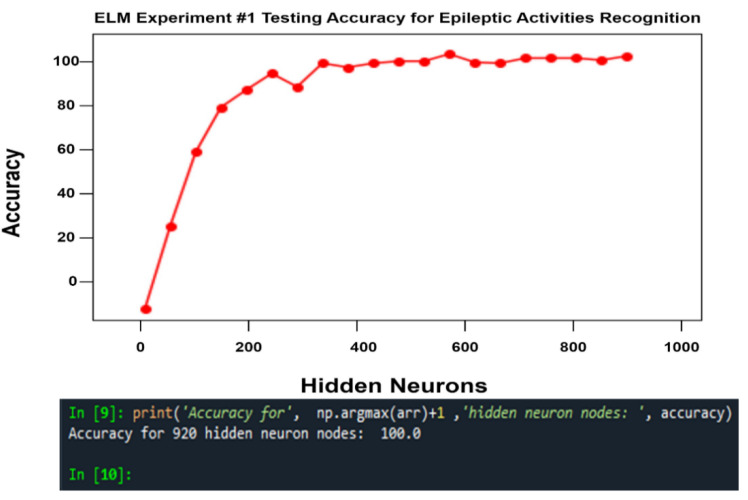
ELM Model Accuracy of Experiment #1.

As can be seen from Table 4, the Extreme Learning Machine yields promising results at this dataset ratio with 920 random hidden neurons, as further shown in Figure 5.

Meanwhile, the random selection of neurons here promises universal approximation, less intervention, global optimal solution, and much faster convergence with less training and testing time. As Figure 5 illustrates, the accuracy of the ELM model increases as the hidden neurons increase in the start, but it gives 100% accuracy at 920 hidden neurons. It is not the case that it always gives the best accuracy just by increasing hidden neurons. The best accuracy is obtained by arbitrarily incremental and random predictions of the given hidden neurons to train itself according to the size of the dataset, following which unimportant hidden neurons are pruned by the model to make it optimal; otherwise, just by increasing the number of hidden neurons in the model, the accuracy will go down due to overfitting.

Another performance evaluation parameter is the ROC curve, which is shown in combined ROC curves in Figure 8, where the best training and testing accuracy of the model is shown for the false positive rate of 1.0 and the true positive rate of 1.0.

As in Figure 9, the confusion matrix chart is shown for and 80% and 20% data partition, where the diagonal matrix shows the correct prediction of positive classes while wrong predictions are shown outside of the diagonal matrix. Here the classifier shows promising results for the ideal prediction of correct classes with no wrong predictions.

### 3.5. Experiment 2

In Experiment 2, epileptic and non-epileptic seizure data are divided into 70% chosen randomly for training and 30% set aside for testing. This ratio achieved 96% accuracy at 805 hidden neuron nodes; other performance parameters are shown in Table 5.

**Figure 6:**
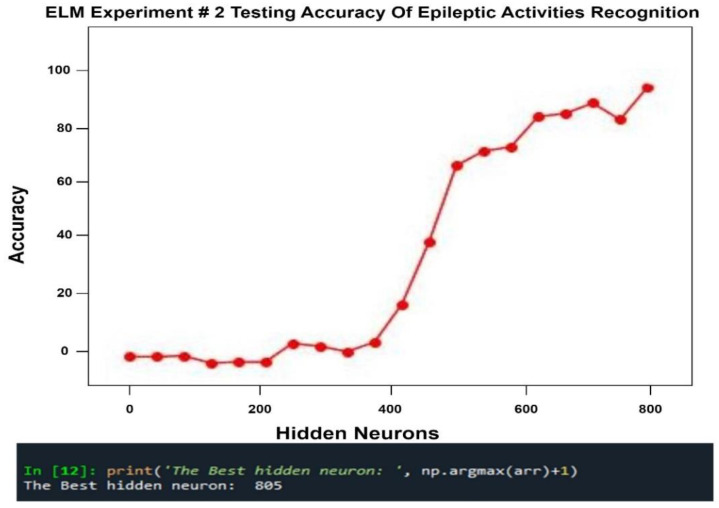
ELM Model Accuracy of Experiment #2.

From Table 5, it can be seen that the Extreme Learning Machine in experiment 2 with 805 random hidden neurons does not yield as promising results as experiment 1, as shown in Figure 6.

From Figure 6, it can be seen that in the start, accuracy is uniform, but as the number of hidden neurons increases, the accuracy is gradually increased, reaching 96% accuracy at best using 805 hidden neuron nodes arbitrarily chosen by the model. Although experiment 2 showed good results, it is not competitive as compared to experiment 1 because the model may become underfit due to the smaller size of the trained dataset and the deep learning model demands huge training datasets for good features training understanding.

As can be seen from Table 5, the ELM at this dataset ratio does not give results as promising as experiment 1. Other performance parameters such as the confusion matrix and ROC curve are shown in Figures 8 and 9, respectively, illustrating that these results are less competitive as compared to experiment 1’s results.

### 3.6. Experiment 3

In experiment 3, the epileptic seizure recognition dataset was randomly divided into 60% for training and 40% for testing purposes, and the maximum accuracy achieved in this partition is 95%. Detailed performance parameters are listed in Table 6**.**

**Figure 7:**
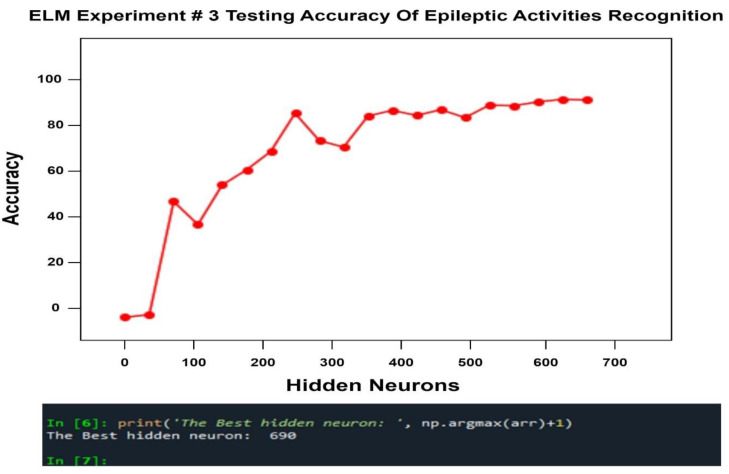
ELM Model Accuracy of Experiment #3.

As seen in Table 6 and Figure 7, the Extreme Learning Machine in experiment 3 with 690 random hidden neurons does not yield as promising results as experiment 1. Experiment 3 also shows much lower values in accuracy and other performance parameters compared to experiment 2. The reason for this is that the same model may become underfit due to lower dataset training ratios.

**Figure 8:**
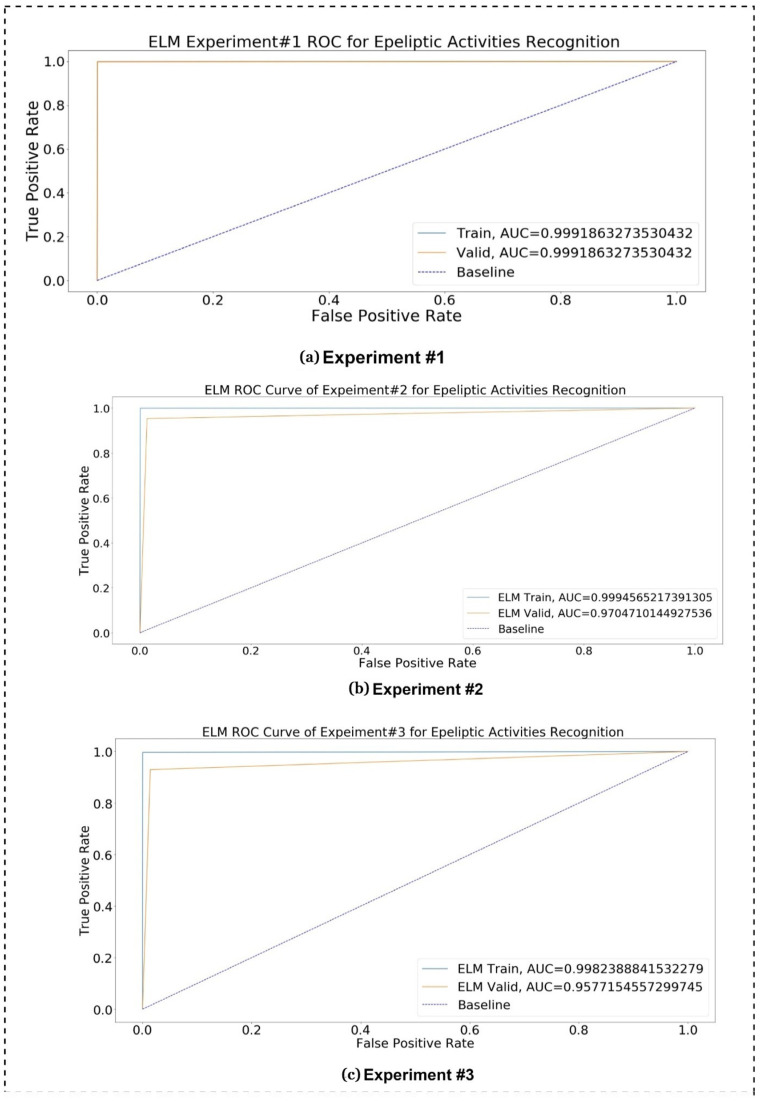
ROC Curves of (**a**) Experiment #1; (**b**) Experiment #2; (**c**) Experiment #3.

**Figure 9:**
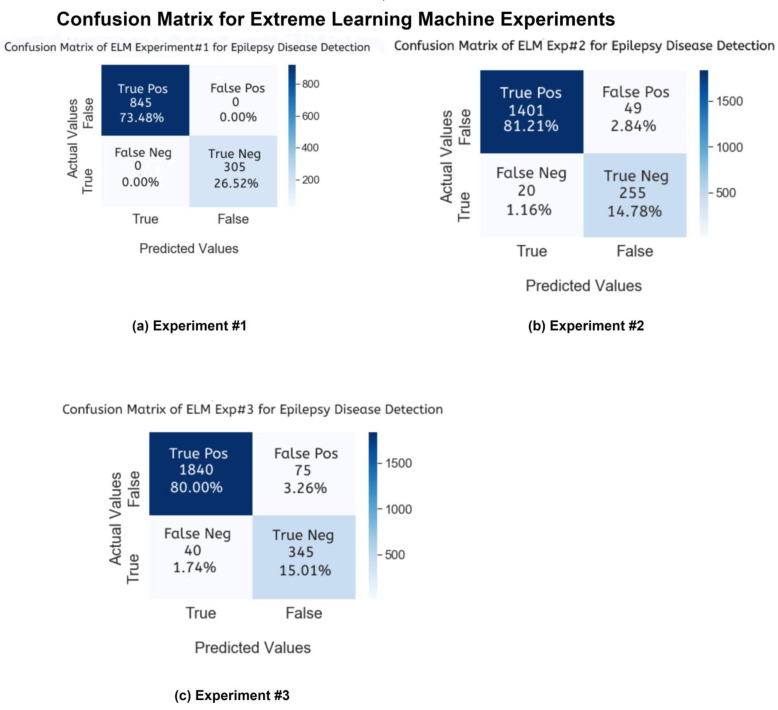
Confusion Matrices of (**a**) Experiment #1; (**b**) Experiment #2; (**c**) Experiment #3.

It can be seen from Figure 7 that accuracy initially uniformly increases as the number of hidden neurons increases, giving the best accuracy of 95% at 690 hidden neuron nodes arbitrarily chosen by the model. Other performance parameters are the confusion matrix chart and ROC curve shown in Figure 8 and Figure 9. Respectively illustrating that these results are less competitive as compared to experiment #1.

## 4. Performance Parameters Comparison of ML and DL Models

In this section, we will report the outcomes of the classifier, named Extreme Learning Machine (ELM), used for the classification of an epilepsy dataset at a ratio of 80% training data with 9200 samples and a 0.500 prevalence of positive classes and 20% testing data with 2300 samples a 0.206 prevalence of positive classes. From this dataset, 10% of the data is set aside for validation and the remaining 10% is used for testing consisting of 1150 samples with a 0.197 prevalence of positive classes for each.

Now it is time to select the classifiers to be compared with the proposed classifier. From previous research results, it is clear that feedforward classifiers are very slow in terms of speed of processing big data, and this issue was the major bottleneck observed in previous research. The reason for this slow speed is the algorithm and classifiers used are low-gradient based, and by using these algorithms, all the required parameters are tuned by the algorithm iteratively. So, keeping in view these reasons, we need a feedforward classifier that can solve these problems. So, the purpose of choosing ELM as a classifier is that it randomly chooses the hidden nodes of its SLFN, and its output weights are determined analytically. The ELM results are enriched in sparsity, stability, and accuracy in general conditions. ELM converges and learns at a very fast speed with good generalization performance when processing big data and does not require user intervention as compared to conventional feedforward algorithms [41].

After keenly observing the previous research we chose the most widely used and competitive machine learning and deep learning models, namely KNN, NB, LR, RF, DT, SGDC, ANN, and LSTM, for comparing with our proposed ELM model using different performance evaluation parameters, namely confusion matrix, accuracy, precision, recall, sensitivity, specificity, F1-score, and AUC.

**Figure 10:**
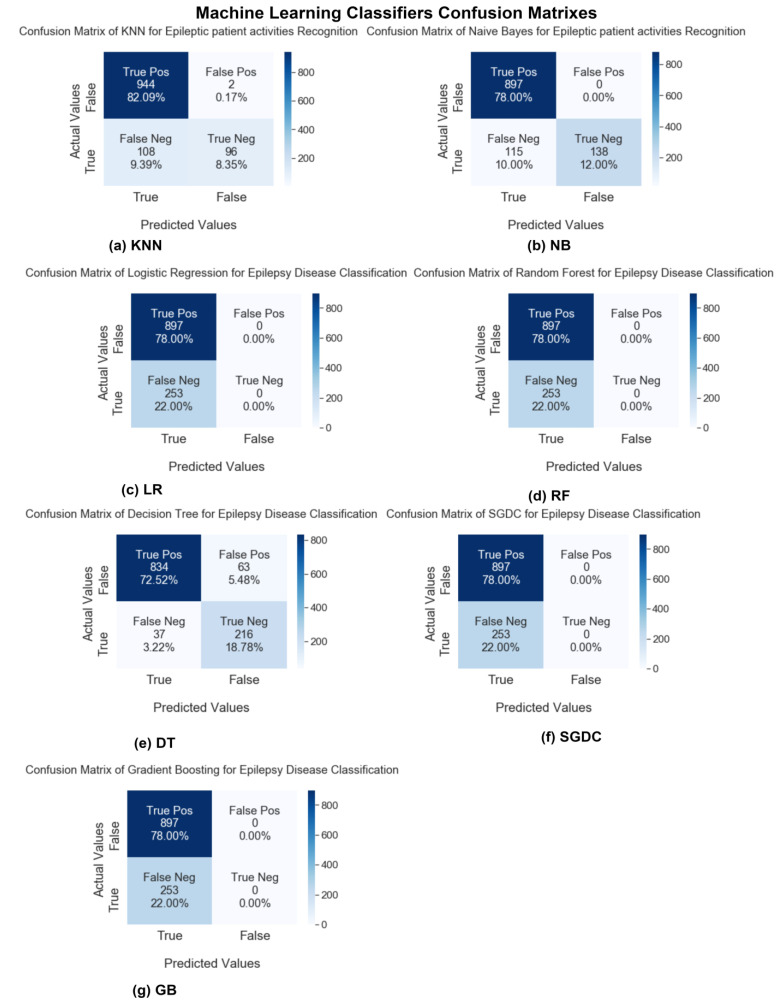
Confusion Matrix of (**a**) KNN, (**b**) NB, (**c**) LR, (**d**) RF, (**e**) DT, (**f**) SGDC, and (**g**) GB.

**Figure 11:**
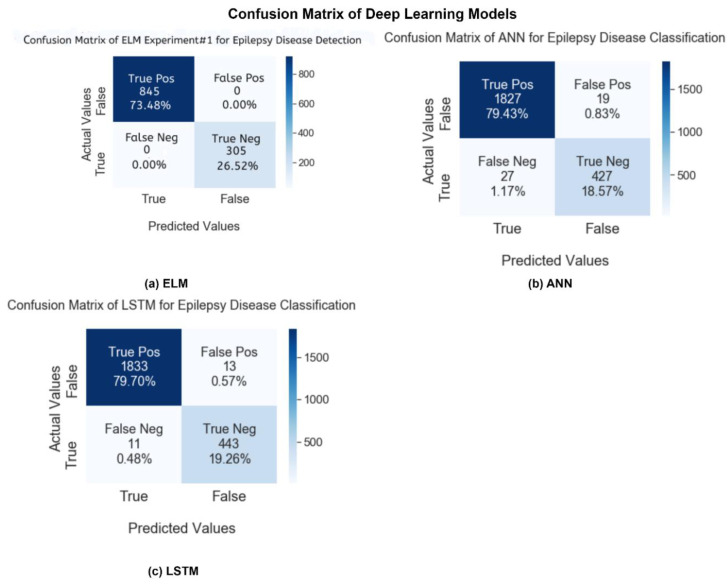
Confusion Matrices of (**a**) ELM #1; (**b**) ANN; (**c**) LSTM.

In this study, after conducting experiments, we will compare the results of our proposed ELM model and other state-of-the-art machine learning and deep learning techniques. First, we will discuss the first performance parameter, the confusion matrix. In Figure 10 and Figure 11, the confusion matrix charts of ML and DL classifiers are represented for epileptic seizure activity recognition, and the column represents the actual label instances of the classes while the rows represent the predicted instances of the actual classes. The correct class count predicted by the model matrix is shown in a diagonal position while the wrong prediction counts of the model are shown outside of the diagonal matrix. Our proposed model, the Extreme Learning Machine (ELM), predicts the wrong predictions and correct predictions with 100% accuracy, as shown in Figure 11.

**Figure 12:**
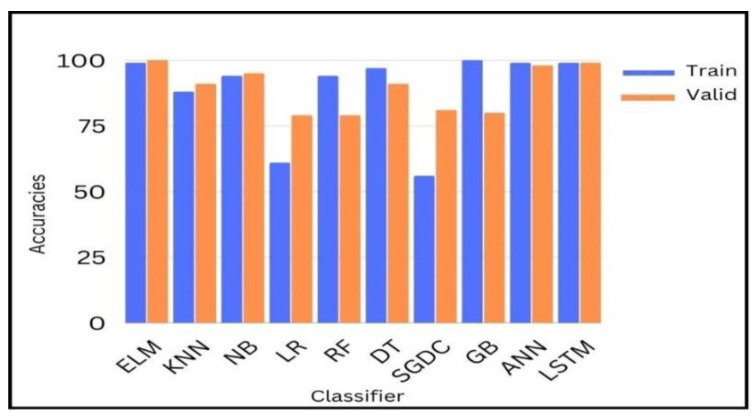
Training and Testing Accuracies of ML and DL Models.

Figure 12 shows the comparison of yielded accuracies of different ML and DL models with our proposed model. Blue bars represent the training accuracies and orange bars represent the testing accuracies of different ML and DL models.

After experiments, we dig deeper into detail to analyze and investigate the result of binary classification by ML and DL classifiers. As can be seen, except for two linear classifiers, logistic regression (LR) and Random Forest (RF), all eight remaining configured classifiers’ results give an accuracy of over 80%, and most of these classifiers show a competitive accuracy of over 90%. In Table 7 our proposed ELM, highlighted in bold, yields the most competitive results as to other models with a low computational time for training and testing.

It is shown that among the ML algorithms, only the Naïve Bayes classifier performed well with a 95% achieved accuracy. A sensitivity analysis was also performed to test the performance of KNN, RF, and SGDC with different parameters, e.g., the training/testing ratio is changed. When the value of K for KNN is changed to above 5, SGDC classifier is used as the regularization parameter, and the loss function is changed then the performance of the classifiers changes accordingly.

After a thorough analysis, it can be seen from Table 7 that among all the classifiers, ELM proved to be high-performing model based on the binary classification technique with a 100% accuracy, 0.99 AUC, 0.95 precision, 0.99 sensitivity and recall, 1.0 specificity, and 0.96 F1-score. The ELM model outperformed other state-of-the-art ML and DL models in the prediction of epileptic and non-epileptic seizures.

This ideal performance of the ELM indicates that it is the best fit for the recognition of epileptic activities; on the other hand, linear models such as LR and RF are not the most suitable classifiers for epileptic seizure activity recognition because the large data requirement (e.g., 11,500 feature) deep learning need for learning and understanding training features effectively will result in overfitting, and these classifiers usually become underfit when using lower training dataset ratios. That is also why our proposed model yields less promising results in experiments 2 and 3 than in our first experiment dataset ratio, i.e., the model is not trained well on low dataset ratios. The ANN and LSTM DL models achieve the nearest accuracies to our model: 98% and 99%, respectively, at the dataset division ratio of 80% and 20%. These models have a low computational testing time but are more time-consuming classifiers for training because they use backpropagation to tune the weights, requiring repetitive iterations and thus more time to train the model. These models also become overfit compared to our proposed model, which has no backpropagation and no iterations to tune the weights. The lack of backpropagation is why a feedforward models converge faster and take less time to train. Due to the absence of backpropagation, our model solves the problem of overfitting experience by other deep learning models. It is worth mentioning here that the power of the best computer workstation is related to the computational time, and it is indirectly indicated that the best workstation has a low computational time. So, it is admitted by our observation that although our model’s results are biased due to the more time-consuming training computational time, the current observed results show that only deep models are fit for epileptic activity recognition tasks as compared to traditional machine learning classifiers.

**Figure 13:**
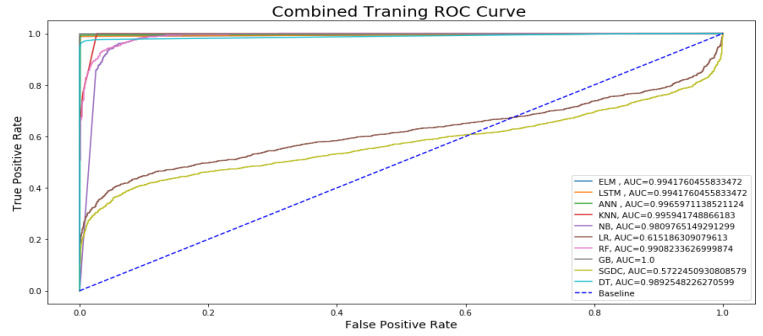
ROC Curve for Training Comparison of Epileptic Activities.

Figure 13 represents the combined training ROC curve of our proposed model in comparison with other state-of-the-art classifiers for epileptic seizure activity recognition. In the training curve, the blue line represents the baseline for the curve. If a model curved at the top left corner of the blue dotted baseline and off to the 45 degree triangle to the top left corner, then it is the best performing model. The curve shows that only two models, namely Logistic Regression (LR) and Stochastic Gradient Descent (SGDC), do not show better performance for the training model, approaching AUC values of 0.61 and 0.57, respectively, because the training line’s accuracy increase at the start and then suddenly starts decreasing and diverges from the baseline and on to the 45 degree triangle. Except for these two models, all other models yield an AUC over 0.90, and the maximum AUC approaches 0.99, yielded by DL models. In the training curve, it can be easily observed that, for training, the model’s true positive rates are higher as compared to the true positive rates for testing. If the figure is closely observed, then it can be concluded that the AUC values of the DL models are higher than those of the ML models for training.

**Figure 14:**
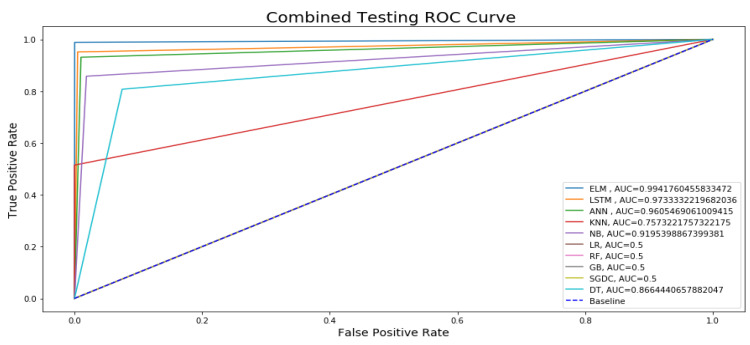
ROC Curve for Testing Comparison of Epileptic Activities Recognition.

Similarly, Figure 14 presents the testing ROC curve of our proposed ELM model with other state-of-the-art ML and DL models. After keenly analyzing the results, it is concluded that only four Machine Learning (ML) models, namely Logistic Regression (LR), Random Forest (RF), Gradient Boosting (GB), and Stochastic Gradient Descent (SGDC), are approaching an AUC of 0.5, and the testing accuracy line following the blue dotted baseline and on to the 45 degree which is not a representation of best-performing models because best performing models are curved at the top of the left corner and off to the 45 degree triangle. All six remaining models are approaching an AUC over 0.70, and the maximum testing AUC of 0.99 is approached by DL models. From the figure, it can be easily observed that the True Positive Rate for the ELM is higher than the other state-of-the-art ML and DL classification techniques. If the testing ROC is closely observed in comparison with the ROC curve of training, then it can be seen that the rate of the training dataset is slightly higher than the testing rates.

## 5. Conclusions

In this research, a deep learning-based technique is applied named Extreme Learning Machine. Some deficiencies in the previous research are overcome in the presented research. Although many machine learning and deep learning techniques have been applied in previous research, there is still more room to improve these models’ accuracy in detecting epilepsy through epileptic activities, and the overfitting problem also needed to be resolved. We improved these imperfections in the presented research. In this research, different Machine Learning algorithms, namely K-Nearest Neighbors (KNN), Logistic Regression (LR), Random Forest (RF), Naïve Bayes (NB), Support Vector Machine (SVM), Stochastic Gradient Descent (SGDC), and Gradient Boosting Classifier (GB), Decision Tree (DT), and Deep Learning (DL) algorithms, namely Long Short-Term Memory (LSTM) and Artificial Neural Network (ANN), are applied. We obtained robust results with the 80% to 20% training to testing ratios. We found that among the ML algorithms, only the Naïve Bayes classifier performed well with a 95% accuracy. Although this ML classifier performed well, the Deep Learning (DL) models are preferred over ML classifiers because of their automated feature extraction and the higher accuracy achieved. The ELM, LSTM, and ANN DL models were used and achieved accuracies of 100%, 99%, and 98%, respectively. Although LSTM and ANN performed well, the accuracy of the ELM is the highest achieved accuracy out of all the models proposed in the current and previous research. This research did not use imaging data to train the classifier, which reduced memory usage compared to using numerical datasets. The model used also does not require backpropagation and thus needs less user intervention. We also tried to overcome the problem of generalization and overfitting faced by the different models by using the ELM classifier. Therefore, it is concluded that the results of this research can be effectively implemented by the community working on the research of epilepsy. We did not check this proposed model for other problems diagnosed using EEG signals from this dataset, but if they lie within the parameters constraints of the model, with a little bit of modification according to the problem’s domain, it should be able to its promising performance for the detection of the problem. The occurrence of epilepsy can be predicted through the detection of epileptic and non-epileptic physical activities instead of through EEG signals collected from the scalp directly or via brain MRI. This research will help neurologists in epilepsy detection and other problems using EEG data by reducing examination time and promising high efficiency and effectiveness.

## Figures and Tables

**Table 1:** Details of the Epileptic Dataset Classes.

Classes Name	No of Samples	Output Classes Labels (Medically)	Description of Classes
1	2300	ictal	Signals recorded during seizures
2	2300	Pre_ictal	Signals recorded before the occurrence of one-site seizure
3	2300	Inter_ictal	Signals recorded during the occurrence of consecutive seizures
4	2300	healthy (close eye)	A healthy subject having closed eyes
5	2300	healthy (open eye)	A healthy subject having open eyes

**Table 2:** Details of the Epileptic Seizure Dataset.

Output Classes	Total No of Records	Normal Records	Abnormal Records	Attributes	Description
5	11,500	9200	2300	X1 to X178	These columns consist of the EEG data of epileptic patients ranging from −1415 to 2047; these variables are called explanatory variables
				X179	In this column, variable y comprises the response variable, and if its value is 0 values, then no seizure is occurring; if the value is 1, then an epileptic seizure is occurring.

**Table 3:** Tuning Parameters of the ELM model.

Parameters	Values
Bias	[0, 1]
Hidden Layers	1
Input Weight Range	[−1, 1]
Output Weight Range	[0, 1]
Input Nodes Size	Arbitrarily Chosen
Activation Function	Sigmoid

**Table 4:** ELM Performance Parameters of Experiment # 1.

ELM Model	Accuracy %	Dataset Division%	Samples	Prevalence	Precision	Recall	F1-Score	Specificity	Sensitivity	AUC
Training	99.9	80	9200	0.500	0.996	0.998	0.996	0.99	0.998	0.999
Testing	100	20	1150	0.19	0.95	0.99	0.969	1.0	0.999	0.999

**Table 5:** ELM Performance Parameters for Experiment # 2.

ELM Model	Accuracy %	Dataset Division%	Samples	Prevalence	Precision	Recall	F1-Score	Specificity	Sensitivity	AUC
Training	99	70	8050	0.500	0.995	0.999	0.996	0.995	0.999	0.999
Testing	96	30	1725	0.206	0.860	0.972	0.912	0.959	0.972	0.970

**Table 6:** ELM Performance Parameters for Experiment # 3.

ELM Model	Accuracy %	Dataset Division%	Samples	Prevalence	Precision	Recall	F1-Score	Specificity	Sensitivity	AUC
Training	99	60	6900	0.500	0.997	0.999	0.997	0.957	0.999	0.998
Testing	95	40	2300	0.213	0.859	0.965	0.908	1.0	0.965	0.957

**Table 7:** The performance comparison of different models.

	Training/Testing Results	Accuracy %	Precision	Recall	F1-Score	Specificity	Sensitivity	AUC	Time (s)
**ML Techniques**									
KNN	Training	95.5	0.992	0.756	0.858	0.994	0.756	0.997	0.015
	Testing	91	0.9927	0.5596	0.7157	0.9988	0.5596	0.7573	0.29
NB	Training	93.7	0.974	0.898	0.933	0.976	0.898	0.982	2.23
	Testing	95	0.9004	0.8559	0.8776	0.9746	0.8559	0.9195	0.42
LR	Training	83.7	0.761	0.532	0.624	0.832	0.532	0.610	0.97
	Testing	79	0.9004	0.8559	0.8776	1.0	0.01	0.5123	0.07
RF	Training	83.7	0.981	0.907	0.941	0.982	0.907	0.9926	0.92
	Testing	79	0.8661	0.895	0.8423	1.0	0.0	0.5122	0.06
DT	Training	98.1	0.988	0.907	0.892	0.988	0.907	0.97	0.057
	Testing	91	0.7415	0.8389	0.7872	0.9245	0.8389	0.8664	0.0008
SGDC	Training	90	0.90	0.907	0.903	0.996	0.907	0.56	0.97
	Testing	81	0.246	0.506	0.225	0.593	0.506	0.5121	0.77
GB	Training	100	1.0	1.0	1.0	1.0	1.0	0.999	0.014
	Testing	80	0.6666	0.0095	0.0188	0.9987	0.0095	0.5111	0.73
**DL Techniques**									
**ELM**	**Training**	**99.9**	**0.996**	**0.998**	**0.996**	**0.99**	**0.998**	**0.1**	**0.96**
	**Testing**	**100**	**0.95**	**0.99**	**0.969**	**1.0**	**0.999**	**0.998**	**0.0009**
ANN	Training	99	0.996	0vb.998	0.996	0.99	0.998	0.992	6.71
	Testing	98	0.9411	0.9515	0.9463	0.9853	0.9515	0.9965	0.005
LSTM	Training	99	0.996	0.998	0.996	0.99	0.998	0.999	7.52
	Testing	99	0.9799	0.9691	0.9745	0.9951	0.9691	0.9941	0.003

## Data Availability

Not applicable.
